# The Role of Companion Dogs in the VA Maryland Health Care System Whole Health(y) GeroFit Program

**DOI:** 10.3390/ani13193047

**Published:** 2023-09-28

**Authors:** Heidi K. Ortmeyer, Jamie Giffuni, Danielle Etchberger, Leslie Katzel

**Affiliations:** 1 Geriatric Research Education Clinical Center, VA Maryland Health Care System, Baltimore, MD 21201, USA; jamie.giffuni@va.gov (J.G.); danielle.etchberger@va.gov (D.E.); leslie.katzel@va.gov (L.K.); 2 Department of Medicine, University of Maryland School of Medicine, Baltimore, MD 21201, USA

**Keywords:** companion dog, GeroFit, veteran, physical activity, physical function, pet attachment, whole health, accelerometry, human–animal bond

## Abstract

**Simple Summary:**

Gym-based exercise in a group setting promotes health and wellness among veterans. However, some veterans are not inclined to participate in gym-based exercise, and alternative options should be considered. We found that providing a companion dog for three months improved the surveyed veterans’ ability to walk longer distances and stand up from a sitting position in a specific amount of time. The number of steps walked and the amount of time physically active throughout the day also increased. Through questionnaires, we found that the veterans were strongly attached to their dogs, and all the veterans reported that their dogs provided love and affection, companionship, and emotional support and facilitated improved social activity participation. We suggest that having a household dog may be an effective alternative to gym-based exercise to promote health and wellness among veterans.

**Abstract:**

GeroFit is a gym-based exercise program that promotes health and wellness among older sedentary veterans. The aims of the current study were to determine whether providing a companion dog as an alternative to gym-based exercise would similarly affect whole health outcomes. A total of 15 (*n* = 15) veterans (62 ± 11 years of age; 13 of 15 >54 years of age) underwent physical function testing, completed global and whole health questionnaires, and wore an accelerometer for 7 days before (baseline) and 3 months after a dog came into their home. The participants completed the Pet Attachment Scale (PAS), Dog Owner-Specific Quality of Life (DOQOL), and Canine Behavioral Assessment and Research questionnaires at 3 months. Cardiorespiratory endurance, lower body strength, daily steps, and time spent engaging in moderate physical activity all increased compared to the baseline levels. Body weight decreased among veterans whose body mass index was ≥30 (*n* = 11). The PAS and DOQOL scores indicated high attachment and positive effects on quality of life after having a dog in the home, with all veterans agreeing that having a dog improved the number of social activities they performed. We conclude that providing a companion dog to veterans not inclined to participate in gym-based exercise is an effective alternative method of promoting health and wellness in this population.

## 1. Introduction

The Veterans Health Administration (VHA) is the largest integrated health care system in the United States, serving nine million enrolled veterans each year. Approximately 60% of older veterans who receive their care through the VHA do not meet physical activity recommendations [1] and are at an increased risk of developing mobility-related disorders, having impaired functional status compared to national-fitness-testing normative data [2]. In one study, older sedentary veterans had lower cardiorespiratory endurance and reduced lower-body strength compared to a national average derived from community-dwelling older adults [2]. In another study, physical function, physical health, and mental health difficulties were related to loneliness severity among veterans, with 57% of older veterans reporting they felt lonely sometimes or often [3]. Difficulty walking one block has been associated with loneliness and social isolation among older individuals [4], and 25% of older veterans report experiencing social isolation [5].

The GeroFit program offered at the VA Maryland Health Care System (VAMHCS) Geriatric Research Education Clinical Center (GRECC) is a gym-based health promotion and exercise program for older veterans [6,7]. GeroFit was developed at the Durham VA Medical Center and was disseminated and implemented at VAMHCS GRECC in 2013. Veterans that participate in the GeroFit program show significant gains in physical function at 3, 6, and 12 months, including an increased 6 min walk distance, usual gait speed, and 30 s chair stands, that are maintained over 5 years of follow-up [7]. More recently, the VHA has shifted to a model of patient-centered care called whole health, which focuses on proactive, personalized health promotion and disease prevention emphasizing complementary and integrative health practices for veterans of all ages [8]. The whole health model of care encourages a veteran to understand their life’s meaning, their aspirations, and their life’s purpose (MAP) and take charge of their life and healthcare, with their MAP as the basis of their decision making [9]. GeroFit has been recognized as a VHA Whole Health program. However, exercise in a traditional gym-setting is not important to every veteran, and as such, they do not increase their physical activity in this way.

We recently completed a feasibility study to determine whether there was an interest among our veteran population in fostering companion dogs, with the aims of increasing physical function and daily activity and improving quality of life [10]. Previous studies have shown the beneficial effects of dog walking on meeting physical activity requirements [11] and on mobility capacity, including increased usual and rapid walking speeds compared to non-walkers [12] among older adults. Dog walking is also beneficial for dogs [13]. For example, obese and non-obese dogs’ daily step counts are inversely related to their body condition scores, suggesting that dogs that walk more often or for longer periods of time have a more favorable body condition score [14]. We found that a two-month companion dog foster program improved physical function (6 min walk distance) and increased daily physical activity (daily steps and moderate activity) among older veterans [10]. In addition, questionnaires and interviews showed the program had positive impacts on the veterans’ well-being and quality of life. Because of these encouraging preliminary results, we added an option for companion dog fostering and adoption to our ongoing GeroFit program to not only improve physical function and increase daily physical activity but also potentially address the negative consequences of social isolation and loneliness. The option to choose a companion dog over gym-based exercise program is in keeping with the mission of VA Whole Health, which is to empower veterans to take charge of their health and focus on what matters to them.

## 2. Materials and Methods

Participants for the companion dog clinical demonstration project were recruited through the GeroFit program at the Baltimore VA Medical Center between 2021 and 2022. Veterans were referred for participation in the program by any VA provider; however, subsequent VA primary care provider approval was necessary. Veterans’ medical histories were reviewed by exercise physiologists in accordance with the program eligibility criteria and screened using the American College of Sports Medicine Preparticipation Health-Screening standards [15]. To qualify for the companion dog program, veterans were required to (a) be medically stable; (b) be able to complete activities of daily living and function independently in a group setting; (c) be willing to commute and provide their own transportation; (d) be free from cognitive impairment, unstable angina, proliferative diabetic retinopathy, oxygen dependency, frank incontinence, open wounds, and/or active substance abuse; (e) not currently have a dog living in their home; (f) be able to properly care for a dog and safely oversee the dog’s exercise; and (g) have received care through the VAMHCS. Veterans then completed a telephone consultation with an exercise physiologist to determine their current exercise status, physical limitations, personal likes and dislikes, and potential barriers to exercise participation or walking a dog. Veterans were then scheduled for a baseline physical function assessment, described below.

Research procedures were approved by the University of Maryland Institutional Research Board (HP-97664) and the Veterans Affairs Research and Development Committee (1651218); all research participants signed informed consent prior to the study’s commencement. 

The principal investigator and veteran worked closely with experienced volunteers at Canine Humane Network and other local rescue groups to find a suitable foster dog for each veteran. The veterans were asked about their previous experiences with dogs; whether they had age, size, and gender preferences; whether there were any types of dogs they would not be comfortable having in their home; and whether they were willing and able to walk their dog for at least 30 min per day. Once a suitable dog(s) had been identified, the veteran and principal investigator met the dog at the home it was currently living in (rescue group foster home) to determine whether the veteran was interested in fostering the dog. Veterans were encouraged to foster dogs that were at least nine months of age so the dogs could sufficiently recover from neuter or spay surgery and be more likely to be housetrained before being sent to a veteran’s home. The participants were informed that (a) they could adopt their foster dog after the 3-month study period was completed (with no adoption fee and a minimum of 12 months of dog food and preventive medicine provided), (b) they would be otherwise financially responsible for the dog if they chose to adopt it, and (c) the participating rescue groups would take their dogs back to the rescue group if for any reason the adopter could no longer care for the dog, for the remainder of the dog’s life. All veterinary expenses, preventive medicines, dog food, and supplies (crate, leash, collar, harness, etc.) were provided during the foster period. All veterinary appointments during the research study were arranged and carried out by the principal investigator. Dog trainers (Maniac Mutts, Hanover, MD, USA) and dog-training classes at PetSmart were made available for the participants throughout the entire period as needed at no cost to the veteran.

At a baseline visit at the Baltimore VA Medical Center, body weight, height, waist circumference, and blood pressure measurements were taken, and the participants completed questionnaires and physical function tests including six-minute walk, 30 s chair stands, four-square step test, and usual gait speed test. The baseline quality-of-life questionnaires included the Short Form Health Survey, SF-36 [16], Global Health Scale [17], and an abbreviated Whole Health for Life Personal Health Inventory—Short Form [18]. The participants wore an ActiGraph GT9X Link monitor (ActiGraph, Pensacola, FL, USA) on their ankles during the six-minute walk so that velocity could be measured (100 Hz sample rate). After the initial visit, the participants were sent home with an ActiGraph GT9X Link monitor (sample rate 100 Hz) to be worn on their wrists for 24 h × 7 days. The monitors were set so that they displayed only time and battery level (no activity measures were visible).

The physical function tests; body weight, waist circumference, and blood pressure measurements; and quality-of-life questionnaires were conducted again at the end of the 3-month foster period. In addition, the participants completed the CENSHARE Pet Attachment Scale (PAS) [19], Canine Behavioral Assessment and Research Questionnaire (C-BARQ) [20], and Dog Owner-Specific Quality of Life Questionnaire (DOQOL) [21], which had been modified for foster caregivers with the permission of the authors [10]. The C-BARQ results were entered into the publicly accessible online website of the C-BARQ Project [22]. The scores were generated on the website and downloaded for further analysis. The C-BARQ site provides population averages from approximately 50,000 pet dogs and more than 300 different breeds and cross-breeds for comparison purposes [22]. The patients wore the Link monitors on their wrists for another 7-day period.

ActiGraph data from the wrist-worn monitors (60 s epoch) were downloaded and screened for wear time using ActiLife v6.13.4 software (ActiGraph, Pensacola, FL, USA). Only data from full 24 h days were analyzed. Cut-off points (counts per minute, cpm) were set at sedentary (<100 cpm), light (100–1951 cpm), moderate (1952–5724 cpm), vigorous (5725–9498 cpm), and very vigorous (≥9499 cpm) [23]. ActiGraph data from the ankle-worn monitors (1 s epoch) were downloaded using ActiLife v6.13.4 software. Rapid walking speed velocity (m/s) was calculated during the six-minute walk by averaging consecutive U-turns (30.48 m apart) divided by the corresponding duration.

Group baseline versus 3-month foster period physical characteristics, physical function, and daily physical activity measurements were compared using a paired *t*-test (2-tailed probability). Baseline versus 3-month foster period Likert scores (Global Health Scale and the Whole Health for Life Personal Health Inventory-Short Form) were compared using paired sign test (2-tailed probability). Spearman’s rank correlations were used to assess relationships between dog-related questionnaire (PAS and DOQOL) scores at 3 months (two-tailed probability). The PAS questionnaire contains five negative (lack-of-attachment) questions (2, 13, 19, 20, and 27), which were reverse-scored before the analyses. Corrections for multiple comparisons were not performed. All data are presented as mean ± SD, with statistical significance set at *p* < 0.05.

## 3. Results

Forty-five veterans were referred to the program through GeroFit either by a VAMHCS provider (primary care, mental health), through self-referral after seeing a program flyer, or through word of mouth. Although traditional GeroFit has a minimum age requirement of 65 years, we allowed veterans of all ages to participate if otherwise eligible. The reasoning behind this decision related to the potential negative effects of the ongoing pandemic on physical activity, mental health, social isolation, and loneliness in our veteran population, and we did not want to turn away any veteran that desired companionship during this time. Twenty veterans were eligible for the companion-dog-fostering program and consented to be in the research study. Two of these veterans had a foster dog in their homes for only three days before deciding they did not want to have a dog in their homes, one veteran was lost to follow-up after baseline testing, one veteran was put on hold after baseline testing due to mental health issues, and one veteran was put on hold after baseline testing due to housing issues. None of the veterans in the research study were concurrently participating in the gym-based group.

Thirteen veterans were matched with dogs (≥9 months old; nine female and four male) through rescue groups. The rescue dogs were altered (neutered or spayed) before the 3-month testing period. Two veterans wanted puppies (female, 10–12 weeks old) and acquired them on their own. Twelve rescue dogs were mixed-breed, including Shepherd, Labrador Retriever, Poodle, Terrier, Border Collie, Pyrenees, and Shiba Inu mixes. One rescue dog was a purebred French Bulldog, and the two puppies were Labrador Retriever and Staffordshire Terrier purebred dogs.

All 15 veterans completed the baseline and 3-month testing. The 11 male and 4 female participants (7 Black, 7 White, and 1 Asian American) ranged between 33–75 (62 ± 11) years of age. Thirteen of the fifteen participants were >54 years of age at baseline. The participants’ physical and metabolic characteristics at baseline and after having a dog in their homes for 3 months are shown in Table 1.

There were no significant changes between the baseline and 3-month values; however, body weight (−2% change, *p* = 0.06) and systolic blood pressure (−6% change, *p* = 0.06) showed a decreasing trend. The participants with a BMI ≥ 30 had decreased body weight following the 3-month foster period (257 ± 29 vs. 250 ± 25 lbs., *p* < 0.05, *n* = 11).

The average baseline and 3-month Global Health Scale and Whole Health questionnaire scores are provided in Table 2. Higher scores are more favorable (5 = “excellent”; 1 = “poor”). The physical health combined score includes physical health, physical performance, and stamina. The mental health combined score includes quality of life, mental health, satisfaction with social activities, and emotional health. The whole health questions include working the body (“How physically active are you?”), recharge (“How well do you sleep, relax, and recover?”), eating and drinking (“Do you consider your eating habits healthy?”), and power of the mind (“How well do you maintain a positive outlook, healthy relationships, & caring for your mental health?”). There were no significant differences between the baseline and 3-months scores. The scores for social activity performance, “how well you carry out your usual social activities and roles,” tended to be different at baseline and after 3 months (*p* = 0.07). The average pain scores (11-point scale; 10 = “worst imaginable pain”) were not different between the baseline and 3-month values (5.1 ± 3 vs. 5.3 ± 3).

The changes in physical function and daily physical activity are shown in Table 3. The distance covered during a six-minute walk, a measure of cardiorespiratory endurance, increased by 11% following the 3-month foster dog period. Usual gait speed did not increase significantly, although there was a significant 12% increase in rapid walking speed during the six-minute walk following the 3-month foster dog period. The number of chair stands in a 30 s period, a measure of lower-body strength, increased by 20% following the 3-month foster dog period. Compared to the baseline values, there was a 23% increase in daily steps and a 69% increase in time spent engaging in moderate physical activity following the 3-month foster period.

The participants’ responses to the Pet Attachment Scale (PAS) questionnaire at 3 months are shown in Table 4. The average PAS score (4-point scale; 4 = almost always) was 3.6 ± 0.3 (*n* = 15). The higher the score, the greater the attachment. Note that questions 2, 13, 19, 20, and 27 were reverse-scored before analysis.

The participants’ responses to the modified Dog Owner-Specific Quality of Life (DOQOL) Questionnaire at 3 months are shown in Table 5 (positive aspects) and Table 6 (negative aspects). 

The average DOQOL score for the positive aspects (7-point scale; 7 = strongly agree) was 6.7 ± 0.5 (*n* = 15). The higher the score, the more potentially beneficial the effect on quality of life. 

The average DOQOL score for the negative aspects (7-point scale; 7 = strongly agree) was 2.1 ± 1.1 (*n* = 15). The higher the score, the greater the potential adverse effect on quality of life. 

The PAS scores were related to the positive aspects of dog ownership (r = 0.56, *p* < 0.05) and inversely related to the negative aspects of dog ownership (r = −0.61, *p* < 0.05).

The veteran, principal investigator, and rescue team worked together to find a well-matched dog based on the veteran’s experiences and living environment. Professional dog trainers and training classes at PetSmart were provided to the veteran free of charge during the 3-month foster period. We asked each veteran to complete the C-BARQ at the 3-month time point in order to (1) obtain an indication of how successful this approach was in choosing dogs with low aggression toward their owners, strangers, and other dogs; (2) determine whether there were remaining behavior issues that needed to be addressed before or after a veteran adopted their dog; and (3) test the relationship between the trainability of the dog and the pet attachment scores. The C-BARQ is a standardized behavioral evaluation tool for dog owners comprising 100 questions grouped into a set of 14 major behavioral traits or factors that describe most of the variation in canine temperament [20]. High scores are less favorable for all behaviors except trainability, for which high scores are more desirable. The study averages ± SD (*n* = 15 dogs) for trainability, 2.49 ± 0.47; chasing, 2.14 ± 1.13; excitability, 1.91 ± 1.02; dog-directed aggression, 1.01 ± 1.04; and dog-directed fear were similar to the population averages [22] (with a study average to population average ratio between 0.95 and 1.04). The study averages for stranger-directed aggression, 0.43 ± 0.44; touch sensitivity, 0.34 ± 0.41; stranger-directed fear, 0.25 ± 0.42; familiar dog aggression, 0.23 ± 0.36; and owner-directed aggression, 0.16 ± 0.31, were lower than the population averages (with a study average to population average ratio between 0.37 and 0.85). The study averages for energy, 2.28 ± 1.28; attachment/attention seeking and nonsocial fear, 1.04 ± 0.81; and separation-related problems, 0.79 ± 1.01, were higher than the population averages (with a study average to population average ratio between 1.16 and 1.42). The trainability (C-BARQ) scores were related to the PAS scores (r = 0.58, *p* < 0.05).

## 4. Discussion

The purpose of the current study was to explore the impact of a novel companion dog foster and adoption program on the physical health and wellness of older veterans, potentially providing an alternative to the ongoing gym-based exercise program. We also examined pet attachment, dog owner-specific quality of life, and the trainability of a dog after a dog and a veteran had lived together for 3 months. The results of this study are encouraging, showing that having a companion dog in the home improved physical function and daily physical activity and that having a companion dog provided emotional and social support in our veteran population.

Numerous cross-sectional studies have reported that dog owners participate in more walking and physical activity compared to those who do not have a dog in their home (as reviewed in [24]). Dog walking, but not dog ownership, for adults ≥50 years of age has been associated with health promotion, including lower BMI values, fewer limitations in activities of daily living, and fewer doctor visits [25]. In the current study, having a dog in the home for 3 months increased the percentage of time spent engaging in moderate physical activity and the number of daily steps taken compared to the baseline (no dog in the home). BMI values decreased 3% among the veterans with a BMI ≥30 at baseline, and systolic blood pressure tended to decrease by 6% in the entire group after having a dog in the home for three months. In a large study of older (71–82-year-old) dog owners, usual and rapid walking speeds were higher than those of non-dog-owners who did not walk at least three times per week and similar to those of non-dog-owners who walked at least 150 min per week [12]. After three years, the dog owners who walked their dogs at baseline were two times as likely to meet recommended walking times of 150 min/week compared to dog owners who did not walk their dogs and to non-dog-owners regardless of whether they walked or did not walk 150 min/week at baseline [12]. In the current study, lower body strength, the distance walked, and a rapid walking speed during the six-minute walk significantly increased for the veterans who had had a dog in their home for three months.

In a recent pilot study, daily physical activity, measured via accelerometry, and heart rate variability were determined to be related among foster caregivers and their foster dogs over a 24 h period [26]. Another cross-sectional study showed dog owners’ BMI was correlated with their dogs’ body condition scores [27]. Lifestyles and interventions that result in higher daily physical activity and lower BMI values among humans may benefit the dogs in their lives. Similarly, programs that provide companion dogs to veterans along with resources including high-quality dog food, veterinary care, supplies (including leashes, harnesses, collars, ID tags, microchips, grooming tools, and car safety belts), preventive medicine, and training are also likely to benefit both humans and dogs. In addition, we suggest that instructions on the importance of daily dog walking, the availability of fresh water, the pros and cons of dog parks, vaccines, heartworm and flea/tick prevention, annual veterinary visits, proper grooming, safety while driving with one’s dog, safe treats and toys, and fall prevention benefit both humans and dogs and strengthen the human–animal bond.

Older veterans present unique experiences of social isolation and loneliness, highlighting the need for programs that address these issues [28]. GeroFit provides a social-support-type club in addition to an exercise component, and social connectedness was highly rated at the 3-month assessment by GeroFit participants over 12 different sites [29]. In the current study, all 15 veterans agreed that fostering a dog improved the number of social activities they performed (DOQOL), and the scores for social activity performance (Global Health Survey) tended to be higher at 3 months compared to the baseline. Whether group exercise or having a companion dog in the home reduces loneliness among veterans is not clear. In a cross-sectional study, veterans with posttraumatic stress disorder had high pet attachment scores and reported feeling less lonely after bringing a dog into their home [30]. In the current study, the scores for satisfaction with social activities and relationships were not different between the baseline measurements and those taken at 3 months. We plan to include additional questions regarding loneliness in future studies to better understand whether companion dogs reduce loneliness in our veteran population. 

The trainability of a dog, as quantified via C-BARQ, is defined as a dog’s willingness to tend to their owner, obey simple commands, learn quickly, fetch objects, respond positively to corrections, and ignore distracting stimuli [22]. In a study of 60 dog-owning families, the trainability of a dog was significantly related to the strength of the owners’ attachments to their dogs (Pet Attachment Scale) [31]. The adults scored their dogs’ trainability at 2.53 ± 0.59 and had PAS scores of 2.8 ± 0.4; the average length of dog ownership was 4.7 ± 2.9 years [31]. In the current study, with the veterans and dogs being together for only three months, the PAS scores were also significantly related to the trainability scores, with similar trainability scores (2.49 ± 0.49) but greater attachment scores (3.6 ± 0.3) compared to the previously mentioned study. Dog owners who scored 100% (*n* = 110) compared to dog owners who scored 0–50% (*n* = 90) on the Dog–Owner Compatibility Index of Activity preferences questionnaire scored their dogs’ trainability significantly higher (2.5 ± 0.7) than the less/non-compatible group (2.0 ± 0.8) [32]. The groups had owned their dogs for an average of 3.1 to 4 years [32].

The limitations of the current study include its small sample size and the lack of a well-matched control group. There were 14 veterans in the gym-based group (with a minimum age of 65) during the same time period as the veterans in the current study, but they were older than the veterans in the companion dog group as we did not want to exclude any veteran from the companion dog program due to age. The comparisons between the baseline measurements and those taken at the 3-month timepoint were not corrected for multiple comparisons, and this could be considered a potential limitation. Another limitation is the lack of a follow-up to determine whether the benefits seen over a three-month period had been maintained over a longer period among those veterans who adopted their foster dogs. To that end, the veterans will be re-tested at 6, 12, and 24 months to determine whether their physical function and mobility have been sustained, whether there have been changes in attachment and dog owner-specific quality of life, and how these changes may relate to changes in dog behavior and the veterans’ physical activity and global and whole health outcomes.

## 5. Conclusions

This study provides promising results for an alternative program to address health and wellness among veterans. We showed that having a companion dog in the home for a three-month foster period promoted physical function, daily physical activity, and quality of life among veterans receiving care through the VHA. The team approach to matching dogs with veterans was successful, as indicated by the high pet attachment and dog owner-specific quality of life scores and well-behaved dogs. This study provides a foundation for a future longitudinal study offering insight into the long-term beneficial effects of companion dogs on the health and wellness of veterans who are not inclined to participate in gym-based exercise programs.

## Figures and Tables

**Table 1:** Physical and metabolic characteristics at baseline and at 3 months.

	Baseline	3-Months
Body weight (lb.)	232 ± 52 (109–307)	227 ± 47 (111–283) ^NS^
Body Mass Index	34 ± 7 (21–51)	33 ± 6 (22–47) ^NS^
Waist circumference (in.)	109 ± 17 (72–129)	109 ± 16 (71–137) ^NS^
Systolic BP (mm Hg)	130 ± 16 (110–173)	121 ± 16 (90–151) ^NS^
Diastolic BP (mm Hg)	76 ± 10 (59–90)	72 ± 13 (54–101) ^NS^

^NS^, not significant vs. Baseline.

**Table 2:** Global and Whole Health questionnaire scores at baseline and 3 months.

**Global Health Scale Questionnaire (Scale 1–5)**		
	**Baseline**	**3-Months**
Physical Health	2.4 ± 0.7	2.5 ± 0.6 ^NS^
Mental Health	1.6 ± 0.5	1.9 ± 0.6 ^NS^
Social Activity Performance	2.8 ± 1.2	3.3 ± 1.3 ^NS^
**Whole Health Questionnaire (Scale 1–5)**		
	**Baseline**	**3-Months**
Physical Activity	2.1 ± 0.9	2.8 ± 0.9 ^NS^
Recharge	2.3 ± 1.0	2.2 ± 1.2 ^NS^
Eating and Drinking	3.0 ± 0.7	3.2 ± 1.0 ^NS^
Power of the Mind	3.1 ± 1.1	3.4 ± 1.2 ^NS^

^NS^, not significant vs. Baseline.

**Table 3:** Physical function and daily physical activity outcomes at baseline and 3 months.

	Baseline	3-Months
Six-minute walk (yards)	422 ± 126	465 ± 140 ^c^
Usual gait speed (m/s)	1.10 ± 0.31	1.16 ± 0.36 ^NS^
Rapid gait speed (m/s)	1.06 ± 0.32	1.19 ± 0.36 ^b^
30 s chair stands (#)	11 ± 4	13 ± 5 ^a^
Daily steps *	10,062 ± 4154	11,551 ± 3639 ^a^
Time engaging in moderate physical activity (%) *	7.2 ± 5	8.8 ± 5.5 ^a^

^a^ *p* < 0.05, ^b^ *p* < 0.005, ^c^ *p* = 0.001 vs. Baseline; ^NS^, not significant vs. Baseline; #, number; * *n* = 14.

**Table 4:** Pet Attachment Scale (CENSHARE).

Question, %	Almost Always	Often	Sometimes	Almost Never
1. Within your family, your pet likes you best.	87	13	0	0
2. You are too busy to spend time with your pet.	0	13	20	67
3. You spend time each day playing with or exercising your pet.	73	27	0	0
4. Your pet comes to greet you when you arrive.	100	0	0	0
5. You talk to your pet as a friend.	93	0	7	0
6. Your pet is aware of your different moods.	53	20	27	0
7.Your pet pays attention and obeys you quickly.	47	13	40	0
8. You confide in your pet.	80	7	0	13
9. You play with your pet when he/she approaches.	73	27	0	0
10. You spend time each day training your pet.	53	20	20	7
11. You show photos of your pet to your friends.	53	33	13	0
12. You spend time each day grooming your pet.	27	53	13	7
13. You ignore your pet when he/she approaches.	0	0	7	93
14. When you come home, your pet is the first one you greet.	73	13	7	7
15. Your pet tries to stay near by following you.	73	27	0	0
16. You buy presents for your pet.	87	7	0	7
17. When you feel bad, you seek your pet for comfort.	73	13	13	0
18. You prefer to be with your pet more than with most people you know.	87	7	7	0
19. When your pet misbehaves, you hit him/her.	0	0	0	100
20. Your pet is a nuisance and a bother to you.	0	0	13	87
21. You consider your pet to be a member of your family.	93	7	0	0
22. You like to touch and stroke your pet.	93	7	0	0
23. You feel sad when you are separated from your pet.	47	20	20	13
24. You like to have your pet sleep in your bed.	33	20	13	33
25. You like to have your pet sleep on your bed.	36	14	7	43
26. You have your pet near you when you study, read, or watch TV.	87	7	7	0
27. You don’t like your pet to get too close to you.	0	0	0	100

**Table 5:** Modified Dog Owner-Specific Quality of Life (DOQOL) Questionnaire: positive aspects.

Question, %	Strongly Agree	Mostly Agree	Somewhat Agree	Neither Agree nor Disagree	Somewhat Disagree	Mostly Disagree	Strongly Disagree
1. Fostering a dog provides me love and affection	87	7	7	0	0	0	0
2. Fostering a dog provides me companionship when I want it	80	7	13	0	0	0	0
3. Fostering a dog provides me emotional support	80	20	0	0	0	0	0
4. Fostering a dog improves the amount of social activities I perform	87	7	7	0	0	0	0
5. Fostering a dog improves my ability to do things for fun outside my home	60	13	20	7	0	0	0
6. Fostering a dog improves my level of physical activity	80	7	13	0	0	0	0

**Table 6:** Modified Dog Owner-Specific Quality of Life (DOQOL) Questionnaire: negative aspects.

Question, %	Strongly Agree	Mostly Agree	Somewhat Agree	Neither Agree nor Disagree	Somewhat Disagree	Mostly Disagree	Strongly Disagree
1. Fostering a dog interferes with my other household responsibilities	0	0	0	13	7	13	67
2. Fostering a dog results in damage to my belongings or property	0	0	27	7	0	7	60
3. Fostering a dog interferes with my ability to go on vacation or leave my house	7	0	13	13	20	7	40
4. Fostering a dog increases my level of stress	0	0	7	7	0	13	73

## Data Availability

The data presented in this study are available on request from the corresponding author. The data are not publicly available due to confidentiality commitments made to both the VAMHCS R&D Committee and VAMHCS IRB of Records, as mandated by Privacy and Information Security Officers at the Baltimore VA Medical Center facility.
